# Supplementary figures and images for: High-throughput phenotyping analysis of maize at the seedling stage using end-to-end segmentation network (part 2 of 2)
Source: PLoS One. 2021 Jan 12;16(1):e0241528. doi: 10.1371/journal.pone.0241528 (PMC7802938; doi:10.1371/journal.pone.0241528)

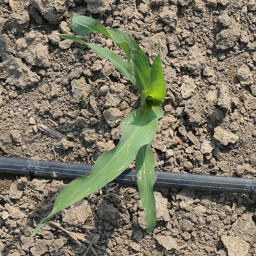

Supplement: S1 File — (ZIP) [file pone.0241528.s001.zip › S1-File/image/74.png]

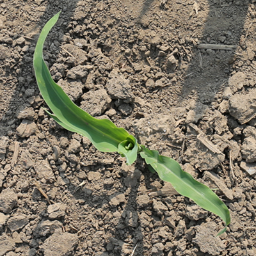

Supplement: S1 File — (ZIP) [file pone.0241528.s001.zip › S1-File/image/75.png]

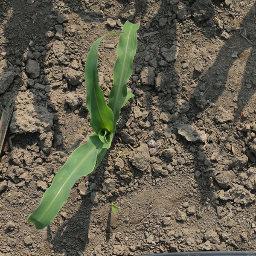

Supplement: S1 File — (ZIP) [file pone.0241528.s001.zip › S1-File/image/76.png]

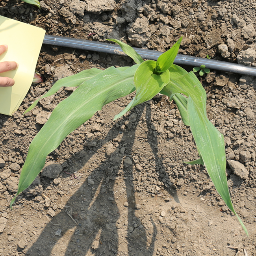

Supplement: S1 File — (ZIP) [file pone.0241528.s001.zip › S1-File/image/77.png]

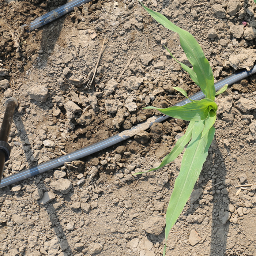

Supplement: S1 File — (ZIP) [file pone.0241528.s001.zip › S1-File/image/78.png]

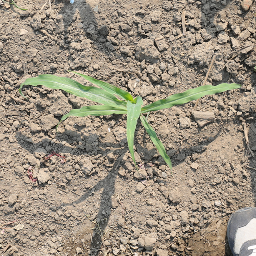

Supplement: S1 File — (ZIP) [file pone.0241528.s001.zip › S1-File/image/79.png]

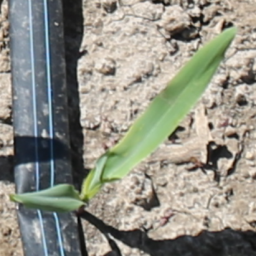

Supplement: S1 File — (ZIP) [file pone.0241528.s001.zip › S1-File/image/8.png]

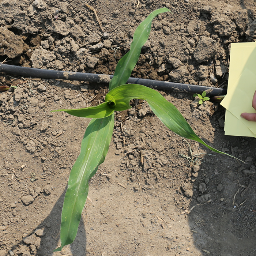

Supplement: S1 File — (ZIP) [file pone.0241528.s001.zip › S1-File/image/80.png]

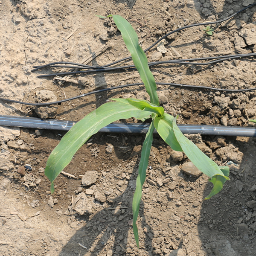

Supplement: S1 File — (ZIP) [file pone.0241528.s001.zip › S1-File/image/81.png]

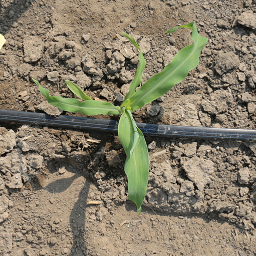

Supplement: S1 File — (ZIP) [file pone.0241528.s001.zip › S1-File/image/82.png]

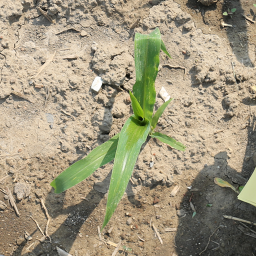

Supplement: S1 File — (ZIP) [file pone.0241528.s001.zip › S1-File/image/83.png]

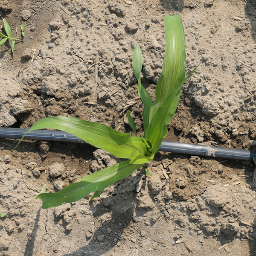

Supplement: S1 File — (ZIP) [file pone.0241528.s001.zip › S1-File/image/84.png]

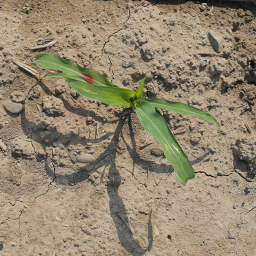

Supplement: S1 File — (ZIP) [file pone.0241528.s001.zip › S1-File/image/85.png]

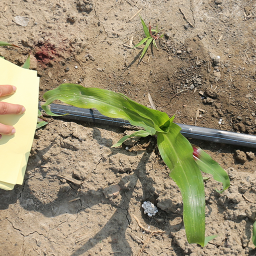

Supplement: S1 File — (ZIP) [file pone.0241528.s001.zip › S1-File/image/86.png]

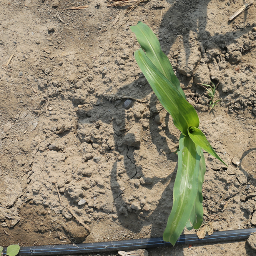

Supplement: S1 File — (ZIP) [file pone.0241528.s001.zip › S1-File/image/87.png]

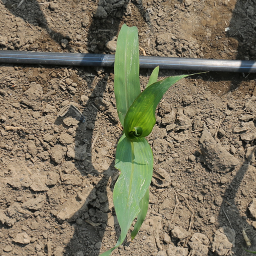

Supplement: S1 File — (ZIP) [file pone.0241528.s001.zip › S1-File/image/88.png]

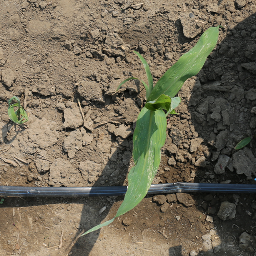

Supplement: S1 File — (ZIP) [file pone.0241528.s001.zip › S1-File/image/89.png]

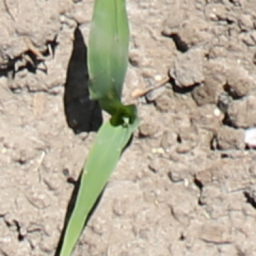

Supplement: S1 File — (ZIP) [file pone.0241528.s001.zip › S1-File/image/9.png]

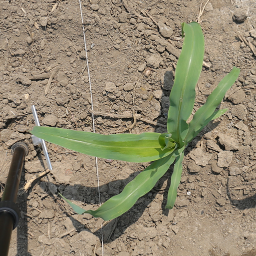

Supplement: S1 File — (ZIP) [file pone.0241528.s001.zip › S1-File/image/90.png]

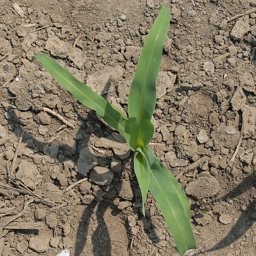

Supplement: S1 File — (ZIP) [file pone.0241528.s001.zip › S1-File/image/91.png]

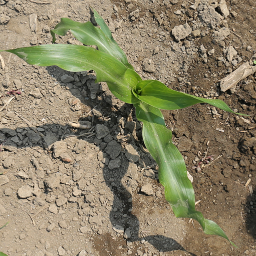

Supplement: S1 File — (ZIP) [file pone.0241528.s001.zip › S1-File/image/92.png]

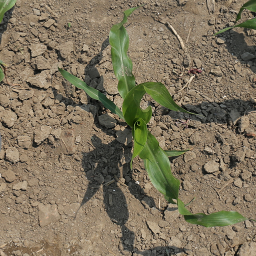

Supplement: S1 File — (ZIP) [file pone.0241528.s001.zip › S1-File/image/93.png]

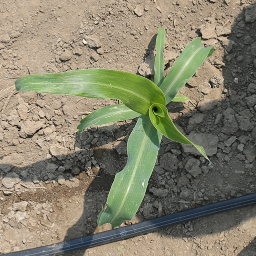

Supplement: S1 File — (ZIP) [file pone.0241528.s001.zip › S1-File/image/94.png]

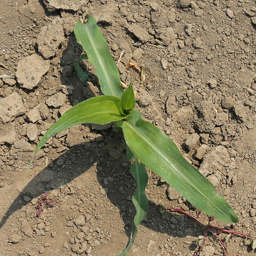

Supplement: S1 File — (ZIP) [file pone.0241528.s001.zip › S1-File/image/95.png]

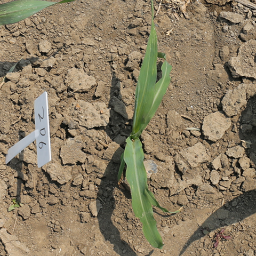

Supplement: S1 File — (ZIP) [file pone.0241528.s001.zip › S1-File/image/96.png]

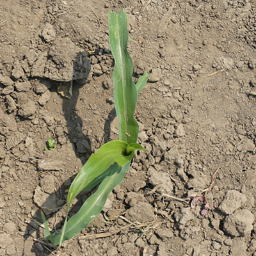

Supplement: S1 File — (ZIP) [file pone.0241528.s001.zip › S1-File/image/97.png]

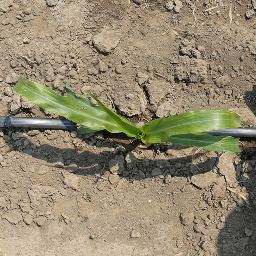

Supplement: S1 File — (ZIP) [file pone.0241528.s001.zip › S1-File/image/98.png]

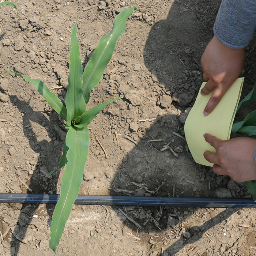

Supplement: S1 File — (ZIP) [file pone.0241528.s001.zip › S1-File/image/99.png]

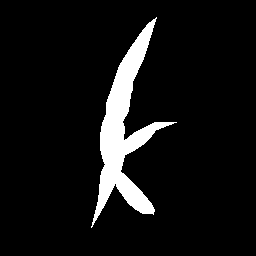

Supplement: S1 File — (ZIP) [file pone.0241528.s001.zip › S1-File/label/1.png]

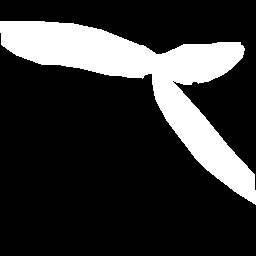

Supplement: S1 File — (ZIP) [file pone.0241528.s001.zip › S1-File/label/10.png]

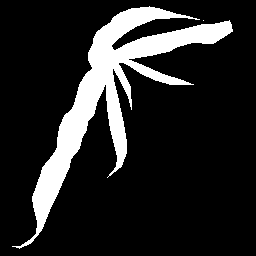

Supplement: S1 File — (ZIP) [file pone.0241528.s001.zip › S1-File/label/100.png]

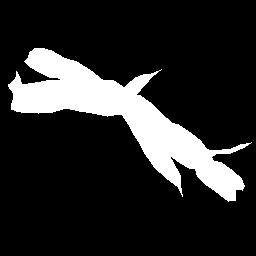

Supplement: S1 File — (ZIP) [file pone.0241528.s001.zip › S1-File/label/101.png]

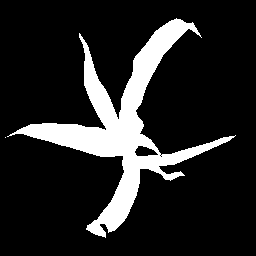

Supplement: S1 File — (ZIP) [file pone.0241528.s001.zip › S1-File/label/102.png]

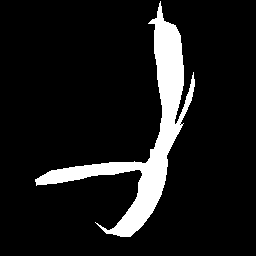

Supplement: S1 File — (ZIP) [file pone.0241528.s001.zip › S1-File/label/103.png]

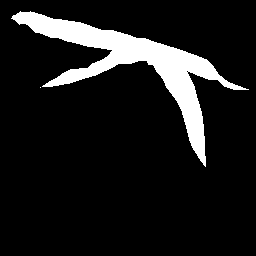

Supplement: S1 File — (ZIP) [file pone.0241528.s001.zip › S1-File/label/104.png]

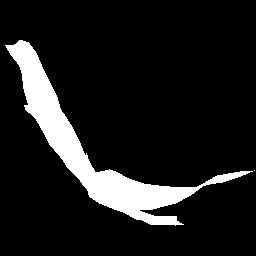

Supplement: S1 File — (ZIP) [file pone.0241528.s001.zip › S1-File/label/105.png]

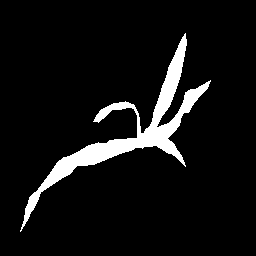

Supplement: S1 File — (ZIP) [file pone.0241528.s001.zip › S1-File/label/106.png]

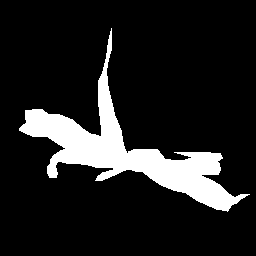

Supplement: S1 File — (ZIP) [file pone.0241528.s001.zip › S1-File/label/107.png]

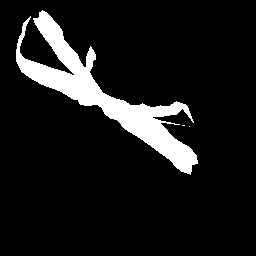

Supplement: S1 File — (ZIP) [file pone.0241528.s001.zip › S1-File/label/108.png]

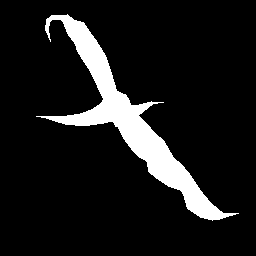

Supplement: S1 File — (ZIP) [file pone.0241528.s001.zip › S1-File/label/109.png]

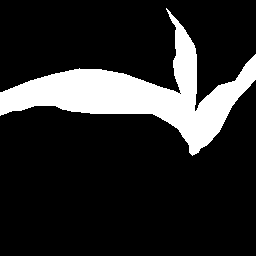

Supplement: S1 File — (ZIP) [file pone.0241528.s001.zip › S1-File/label/11.png]

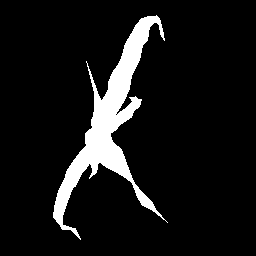

Supplement: S1 File — (ZIP) [file pone.0241528.s001.zip › S1-File/label/110.png]

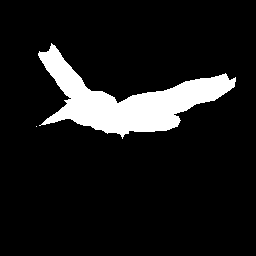

Supplement: S1 File — (ZIP) [file pone.0241528.s001.zip › S1-File/label/111.png]

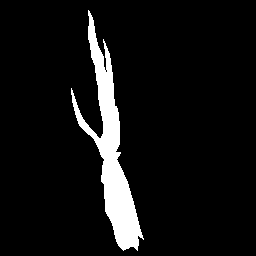

Supplement: S1 File — (ZIP) [file pone.0241528.s001.zip › S1-File/label/112.png]

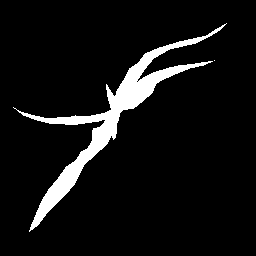

Supplement: S1 File — (ZIP) [file pone.0241528.s001.zip › S1-File/label/113.png]

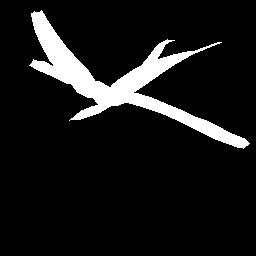

Supplement: S1 File — (ZIP) [file pone.0241528.s001.zip › S1-File/label/114.png]

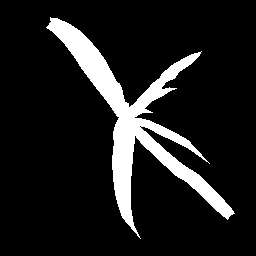

Supplement: S1 File — (ZIP) [file pone.0241528.s001.zip › S1-File/label/115.png]

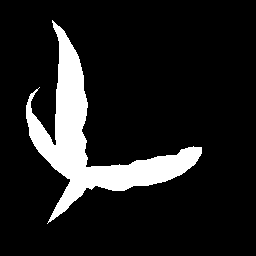

Supplement: S1 File — (ZIP) [file pone.0241528.s001.zip › S1-File/label/116.png]

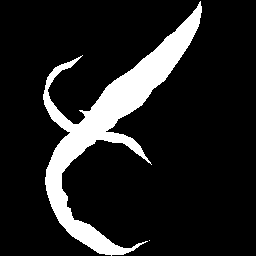

Supplement: S1 File — (ZIP) [file pone.0241528.s001.zip › S1-File/label/117.png]

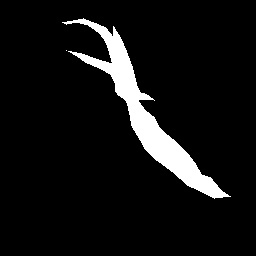

Supplement: S1 File — (ZIP) [file pone.0241528.s001.zip › S1-File/label/118.png]

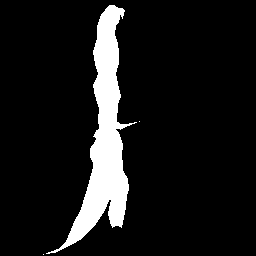

Supplement: S1 File — (ZIP) [file pone.0241528.s001.zip › S1-File/label/119.png]

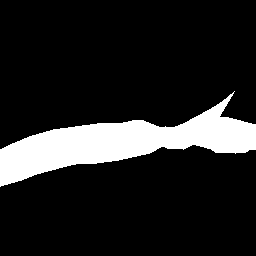

Supplement: S1 File — (ZIP) [file pone.0241528.s001.zip › S1-File/label/12.png]

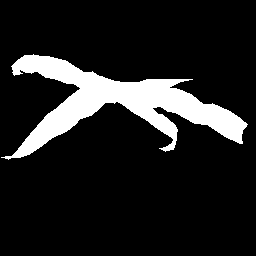

Supplement: S1 File — (ZIP) [file pone.0241528.s001.zip › S1-File/label/120.png]

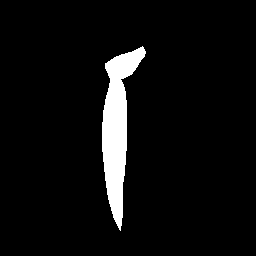

Supplement: S1 File — (ZIP) [file pone.0241528.s001.zip › S1-File/label/121.png]

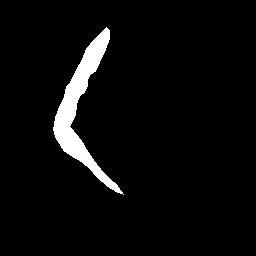

Supplement: S1 File — (ZIP) [file pone.0241528.s001.zip › S1-File/label/122.png]

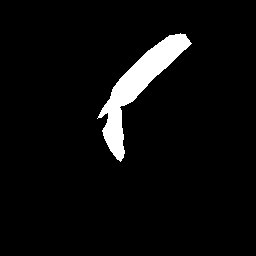

Supplement: S1 File — (ZIP) [file pone.0241528.s001.zip › S1-File/label/123.png]

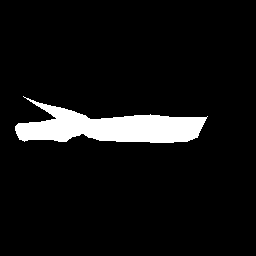

Supplement: S1 File — (ZIP) [file pone.0241528.s001.zip › S1-File/label/124.png]

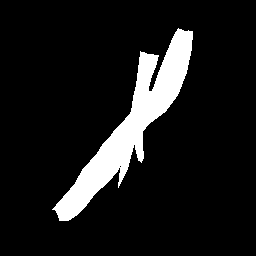

Supplement: S1 File — (ZIP) [file pone.0241528.s001.zip › S1-File/label/125.png]

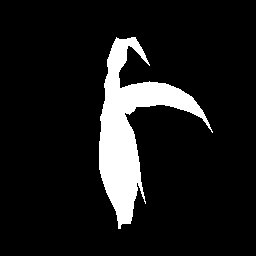

Supplement: S1 File — (ZIP) [file pone.0241528.s001.zip › S1-File/label/126.png]

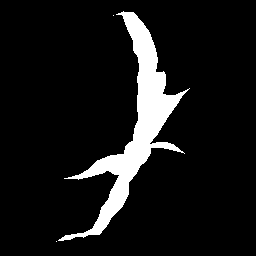

Supplement: S1 File — (ZIP) [file pone.0241528.s001.zip › S1-File/label/127.png]

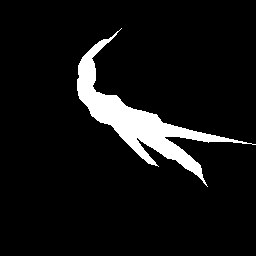

Supplement: S1 File — (ZIP) [file pone.0241528.s001.zip › S1-File/label/128.png]

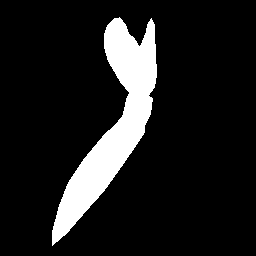

Supplement: S1 File — (ZIP) [file pone.0241528.s001.zip › S1-File/label/13.png]

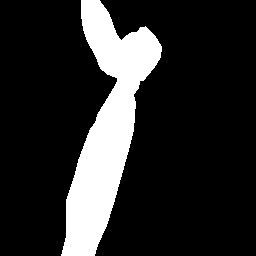

Supplement: S1 File — (ZIP) [file pone.0241528.s001.zip › S1-File/label/14.png]

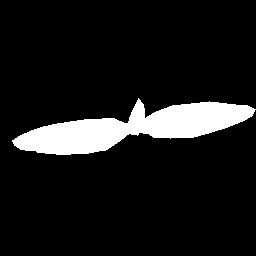

Supplement: S1 File — (ZIP) [file pone.0241528.s001.zip › S1-File/label/15.png]

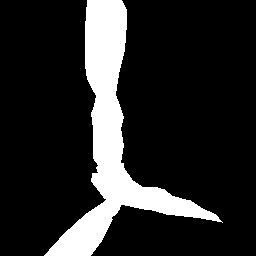

Supplement: S1 File — (ZIP) [file pone.0241528.s001.zip › S1-File/label/16.png]

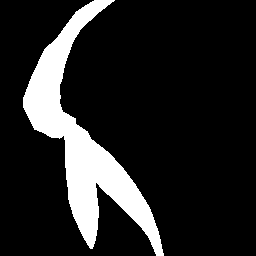

Supplement: S1 File — (ZIP) [file pone.0241528.s001.zip › S1-File/label/17.png]

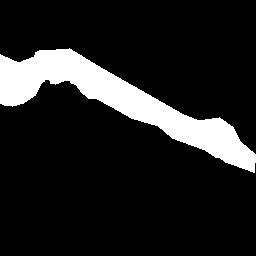

Supplement: S1 File — (ZIP) [file pone.0241528.s001.zip › S1-File/label/18.png]

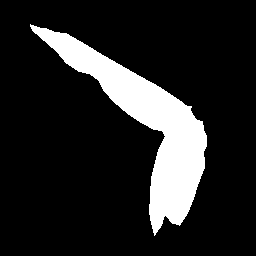

Supplement: S1 File — (ZIP) [file pone.0241528.s001.zip › S1-File/label/19.png]

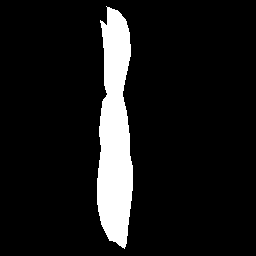

Supplement: S1 File — (ZIP) [file pone.0241528.s001.zip › S1-File/label/2.png]

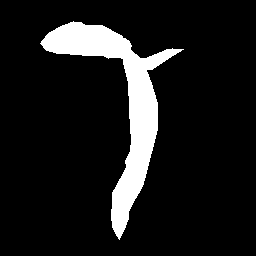

Supplement: S1 File — (ZIP) [file pone.0241528.s001.zip › S1-File/label/20.png]

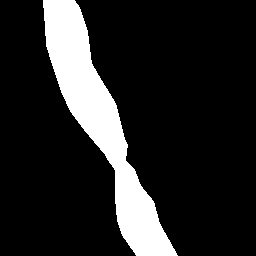

Supplement: S1 File — (ZIP) [file pone.0241528.s001.zip › S1-File/label/21.png]

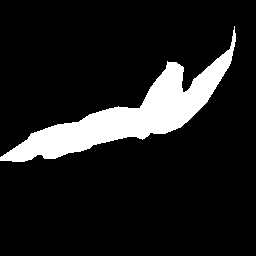

Supplement: S1 File — (ZIP) [file pone.0241528.s001.zip › S1-File/label/22.png]

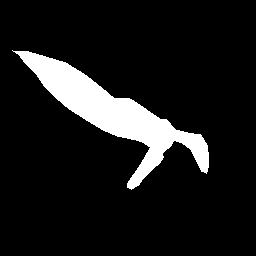

Supplement: S1 File — (ZIP) [file pone.0241528.s001.zip › S1-File/label/23.png]

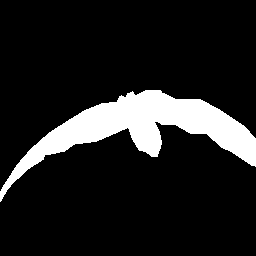

Supplement: S1 File — (ZIP) [file pone.0241528.s001.zip › S1-File/label/24.png]

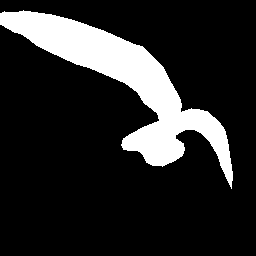

Supplement: S1 File — (ZIP) [file pone.0241528.s001.zip › S1-File/label/25.png]

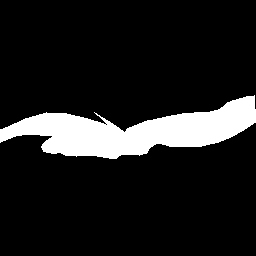

Supplement: S1 File — (ZIP) [file pone.0241528.s001.zip › S1-File/label/26.png]

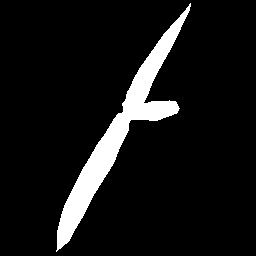

Supplement: S1 File — (ZIP) [file pone.0241528.s001.zip › S1-File/label/27.png]

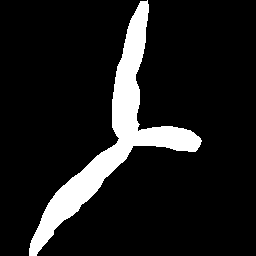

Supplement: S1 File — (ZIP) [file pone.0241528.s001.zip › S1-File/label/28.png]

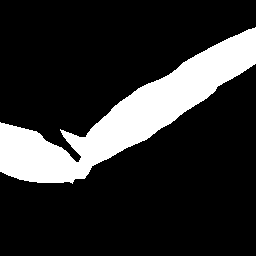

Supplement: S1 File — (ZIP) [file pone.0241528.s001.zip › S1-File/label/29.png]

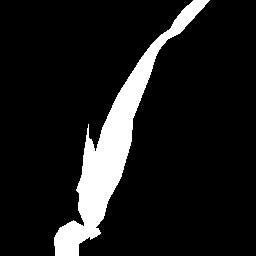

Supplement: S1 File — (ZIP) [file pone.0241528.s001.zip › S1-File/label/3.png]

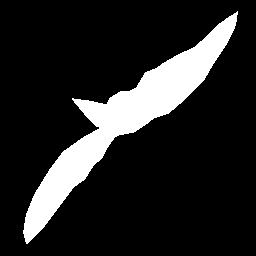

Supplement: S1 File — (ZIP) [file pone.0241528.s001.zip › S1-File/label/30.png]

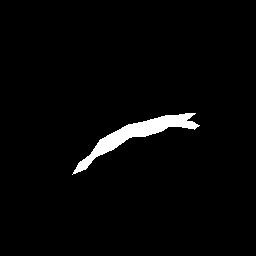

Supplement: S1 File — (ZIP) [file pone.0241528.s001.zip › S1-File/label/31.png]

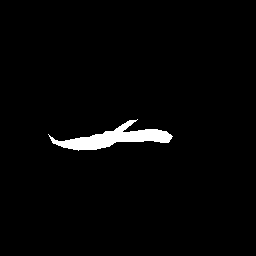

Supplement: S1 File — (ZIP) [file pone.0241528.s001.zip › S1-File/label/32.png]

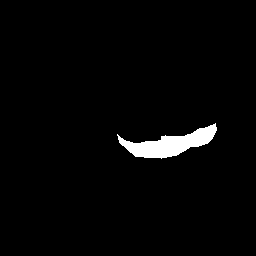

Supplement: S1 File — (ZIP) [file pone.0241528.s001.zip › S1-File/label/33.png]

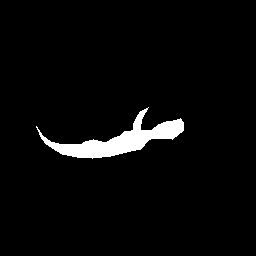

Supplement: S1 File — (ZIP) [file pone.0241528.s001.zip › S1-File/label/34.png]

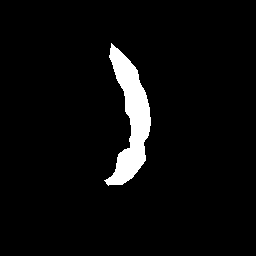

Supplement: S1 File — (ZIP) [file pone.0241528.s001.zip › S1-File/label/35.png]

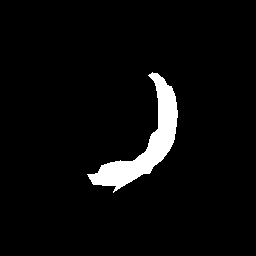

Supplement: S1 File — (ZIP) [file pone.0241528.s001.zip › S1-File/label/36.png]

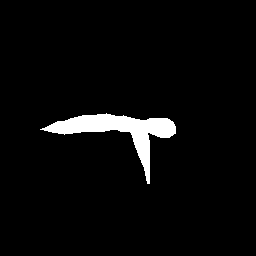

Supplement: S1 File — (ZIP) [file pone.0241528.s001.zip › S1-File/label/37.png]

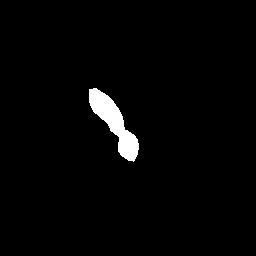

Supplement: S1 File — (ZIP) [file pone.0241528.s001.zip › S1-File/label/38.png]

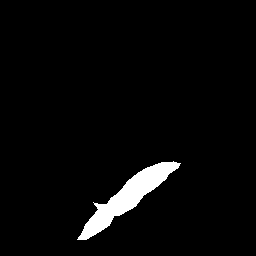

Supplement: S1 File — (ZIP) [file pone.0241528.s001.zip › S1-File/label/39.png]

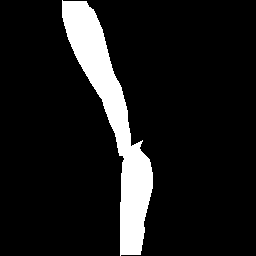

Supplement: S1 File — (ZIP) [file pone.0241528.s001.zip › S1-File/label/4.png]

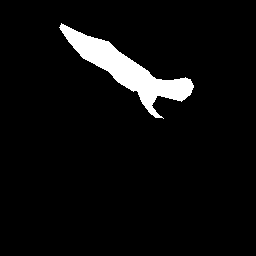

Supplement: S1 File — (ZIP) [file pone.0241528.s001.zip › S1-File/label/40.png]

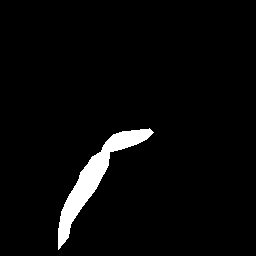

Supplement: S1 File — (ZIP) [file pone.0241528.s001.zip › S1-File/label/41.png]

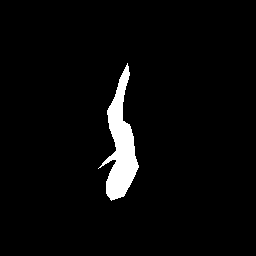

Supplement: S1 File — (ZIP) [file pone.0241528.s001.zip › S1-File/label/42.png]

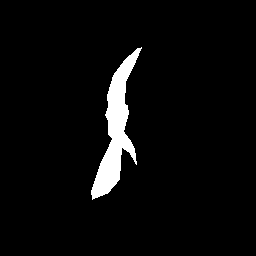

Supplement: S1 File — (ZIP) [file pone.0241528.s001.zip › S1-File/label/43.png]

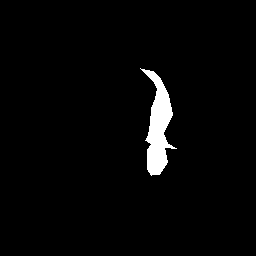

Supplement: S1 File — (ZIP) [file pone.0241528.s001.zip › S1-File/label/44.png]

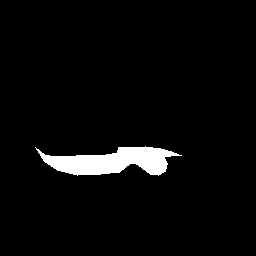

Supplement: S1 File — (ZIP) [file pone.0241528.s001.zip › S1-File/label/45.png]

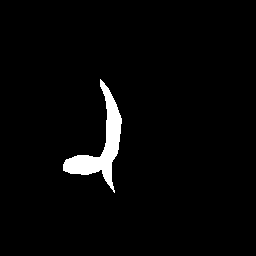

Supplement: S1 File — (ZIP) [file pone.0241528.s001.zip › S1-File/label/46.png]

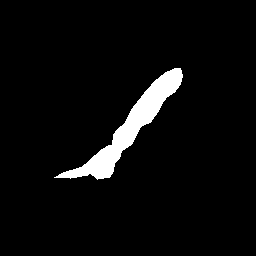

Supplement: S1 File — (ZIP) [file pone.0241528.s001.zip › S1-File/label/47.png]

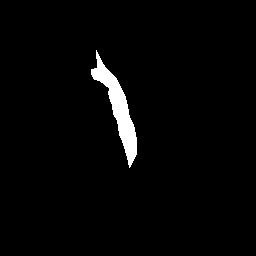

Supplement: S1 File — (ZIP) [file pone.0241528.s001.zip › S1-File/label/48.png]
