# Supplementary figures and images for: High-throughput phenotyping analysis of maize at the seedling stage using end-to-end segmentation network (part 1 of 2)
Source: PLoS One. 2021 Jan 12;16(1):e0241528. doi: 10.1371/journal.pone.0241528 (PMC7802938; doi:10.1371/journal.pone.0241528)

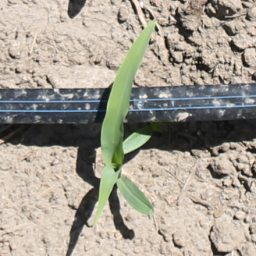

Supplement: S1 File — (ZIP) [file pone.0241528.s001.zip › S1-File/image/1.png]

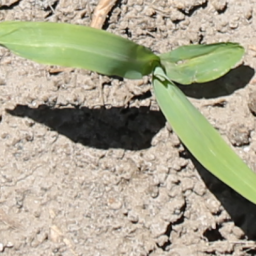

Supplement: S1 File — (ZIP) [file pone.0241528.s001.zip › S1-File/image/10.png]

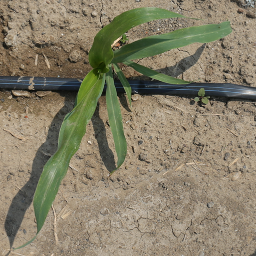

Supplement: S1 File — (ZIP) [file pone.0241528.s001.zip › S1-File/image/100.png]

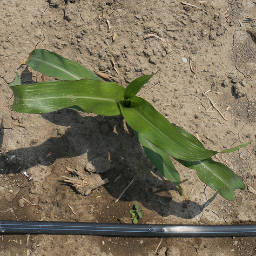

Supplement: S1 File — (ZIP) [file pone.0241528.s001.zip › S1-File/image/101.png]

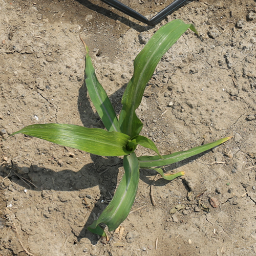

Supplement: S1 File — (ZIP) [file pone.0241528.s001.zip › S1-File/image/102.png]

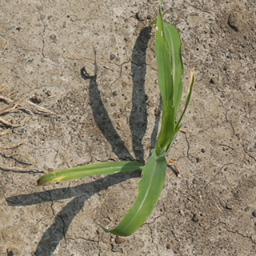

Supplement: S1 File — (ZIP) [file pone.0241528.s001.zip › S1-File/image/103.png]

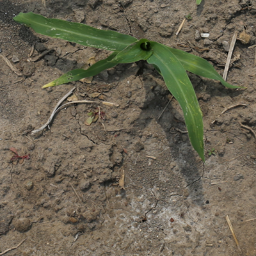

Supplement: S1 File — (ZIP) [file pone.0241528.s001.zip › S1-File/image/104.png]

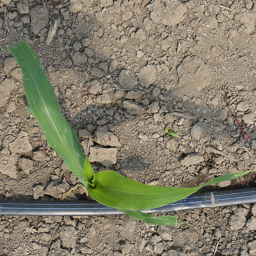

Supplement: S1 File — (ZIP) [file pone.0241528.s001.zip › S1-File/image/105.png]

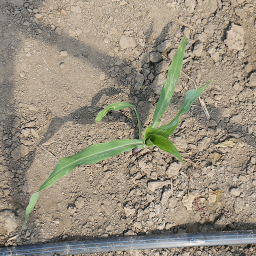

Supplement: S1 File — (ZIP) [file pone.0241528.s001.zip › S1-File/image/106.png]

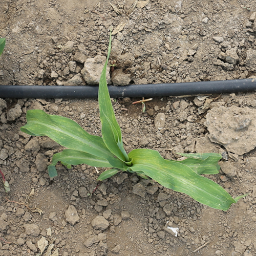

Supplement: S1 File — (ZIP) [file pone.0241528.s001.zip › S1-File/image/107.png]

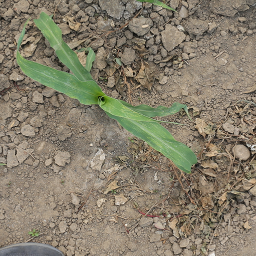

Supplement: S1 File — (ZIP) [file pone.0241528.s001.zip › S1-File/image/108.png]

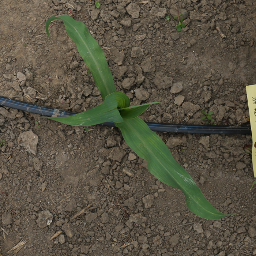

Supplement: S1 File — (ZIP) [file pone.0241528.s001.zip › S1-File/image/109.png]

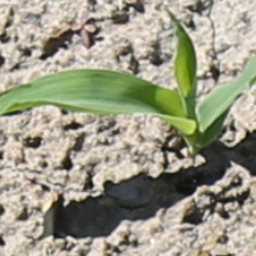

Supplement: S1 File — (ZIP) [file pone.0241528.s001.zip › S1-File/image/11.png]

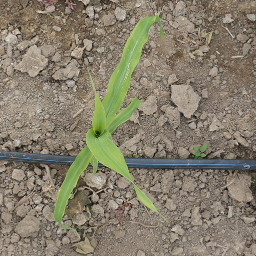

Supplement: S1 File — (ZIP) [file pone.0241528.s001.zip › S1-File/image/110.png]

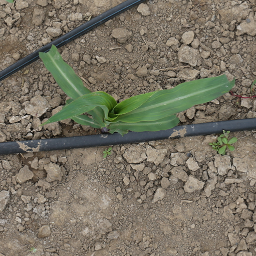

Supplement: S1 File — (ZIP) [file pone.0241528.s001.zip › S1-File/image/111.png]

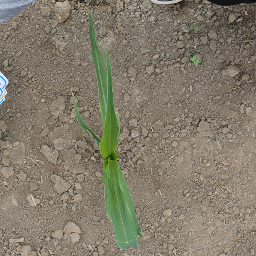

Supplement: S1 File — (ZIP) [file pone.0241528.s001.zip › S1-File/image/112.png]

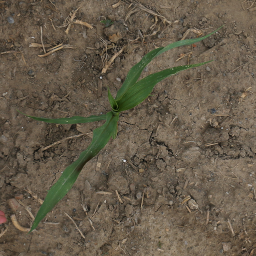

Supplement: S1 File — (ZIP) [file pone.0241528.s001.zip › S1-File/image/113.png]

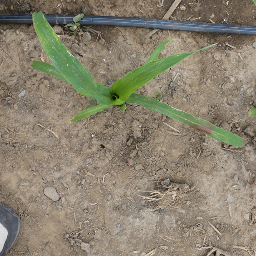

Supplement: S1 File — (ZIP) [file pone.0241528.s001.zip › S1-File/image/114.png]

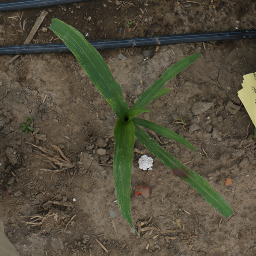

Supplement: S1 File — (ZIP) [file pone.0241528.s001.zip › S1-File/image/115.png]

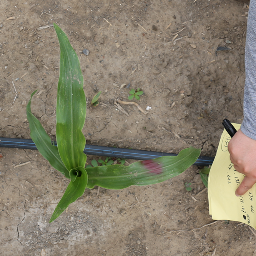

Supplement: S1 File — (ZIP) [file pone.0241528.s001.zip › S1-File/image/116.png]

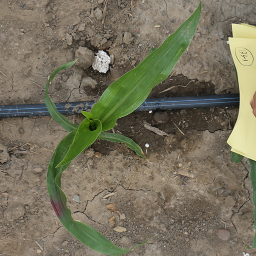

Supplement: S1 File — (ZIP) [file pone.0241528.s001.zip › S1-File/image/117.png]

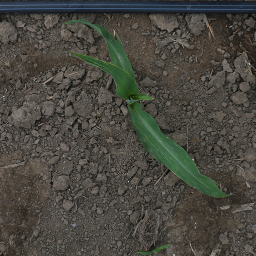

Supplement: S1 File — (ZIP) [file pone.0241528.s001.zip › S1-File/image/118.png]

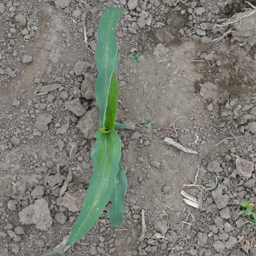

Supplement: S1 File — (ZIP) [file pone.0241528.s001.zip › S1-File/image/119.png]

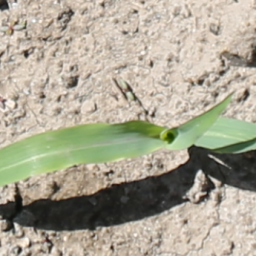

Supplement: S1 File — (ZIP) [file pone.0241528.s001.zip › S1-File/image/12.png]

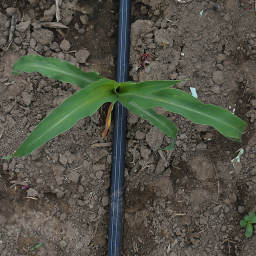

Supplement: S1 File — (ZIP) [file pone.0241528.s001.zip › S1-File/image/120.png]

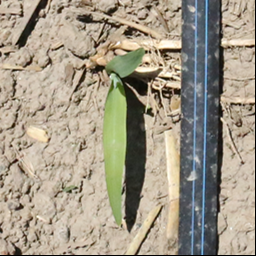

Supplement: S1 File — (ZIP) [file pone.0241528.s001.zip › S1-File/image/121.png]

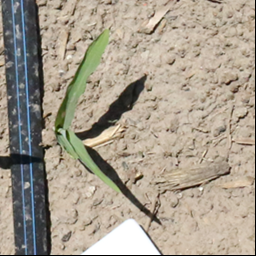

Supplement: S1 File — (ZIP) [file pone.0241528.s001.zip › S1-File/image/122.png]

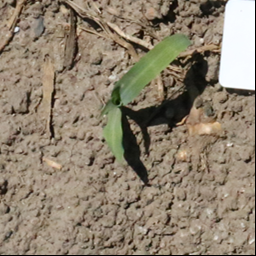

Supplement: S1 File — (ZIP) [file pone.0241528.s001.zip › S1-File/image/123.png]

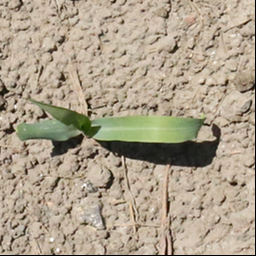

Supplement: S1 File — (ZIP) [file pone.0241528.s001.zip › S1-File/image/124.png]

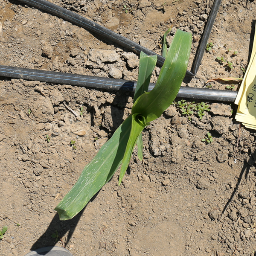

Supplement: S1 File — (ZIP) [file pone.0241528.s001.zip › S1-File/image/125.png]

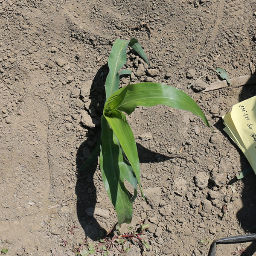

Supplement: S1 File — (ZIP) [file pone.0241528.s001.zip › S1-File/image/126.png]

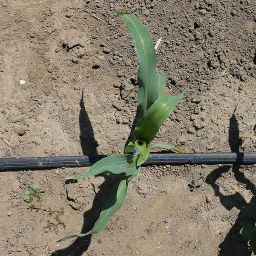

Supplement: S1 File — (ZIP) [file pone.0241528.s001.zip › S1-File/image/127.png]

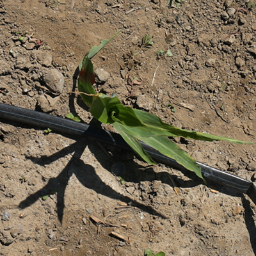

Supplement: S1 File — (ZIP) [file pone.0241528.s001.zip › S1-File/image/128.png]

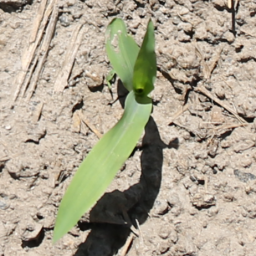

Supplement: S1 File — (ZIP) [file pone.0241528.s001.zip › S1-File/image/13.png]

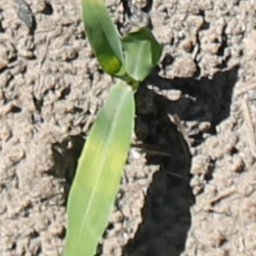

Supplement: S1 File — (ZIP) [file pone.0241528.s001.zip › S1-File/image/14.png]

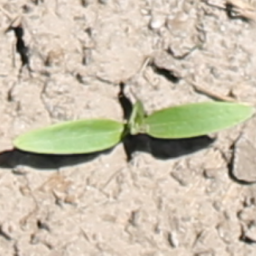

Supplement: S1 File — (ZIP) [file pone.0241528.s001.zip › S1-File/image/15.png]

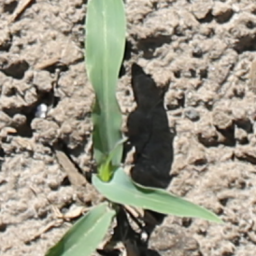

Supplement: S1 File — (ZIP) [file pone.0241528.s001.zip › S1-File/image/16.png]

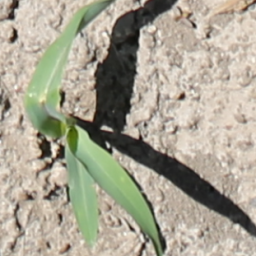

Supplement: S1 File — (ZIP) [file pone.0241528.s001.zip › S1-File/image/17.png]

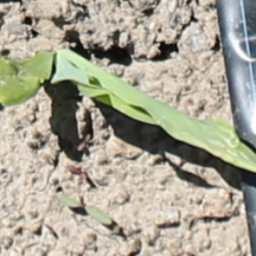

Supplement: S1 File — (ZIP) [file pone.0241528.s001.zip › S1-File/image/18.png]

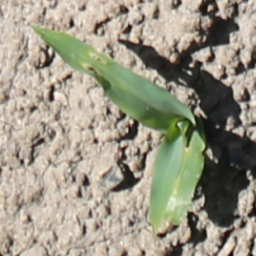

Supplement: S1 File — (ZIP) [file pone.0241528.s001.zip › S1-File/image/19.png]

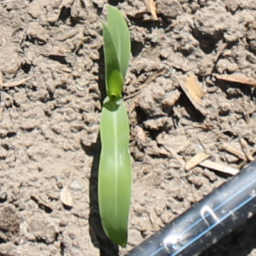

Supplement: S1 File — (ZIP) [file pone.0241528.s001.zip › S1-File/image/2.png]

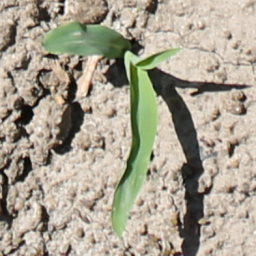

Supplement: S1 File — (ZIP) [file pone.0241528.s001.zip › S1-File/image/20.png]

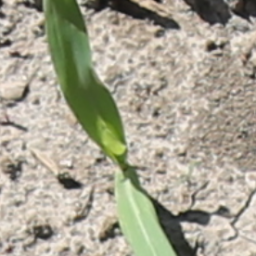

Supplement: S1 File — (ZIP) [file pone.0241528.s001.zip › S1-File/image/21.png]

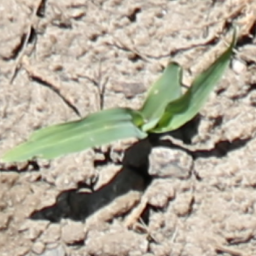

Supplement: S1 File — (ZIP) [file pone.0241528.s001.zip › S1-File/image/22.png]

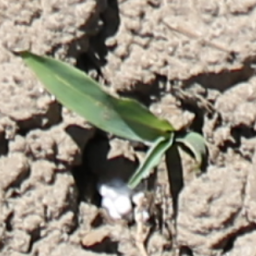

Supplement: S1 File — (ZIP) [file pone.0241528.s001.zip › S1-File/image/23.png]

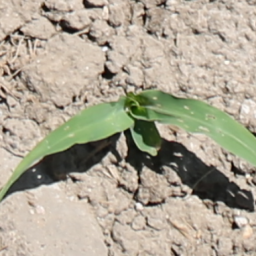

Supplement: S1 File — (ZIP) [file pone.0241528.s001.zip › S1-File/image/24.png]

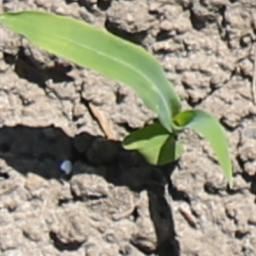

Supplement: S1 File — (ZIP) [file pone.0241528.s001.zip › S1-File/image/25.png]

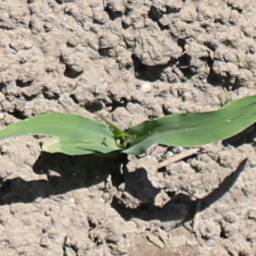

Supplement: S1 File — (ZIP) [file pone.0241528.s001.zip › S1-File/image/26.png]

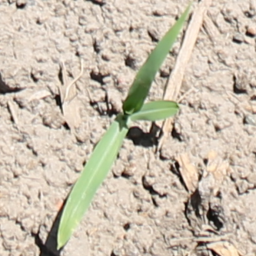

Supplement: S1 File — (ZIP) [file pone.0241528.s001.zip › S1-File/image/27.png]

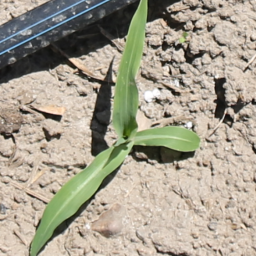

Supplement: S1 File — (ZIP) [file pone.0241528.s001.zip › S1-File/image/28.png]

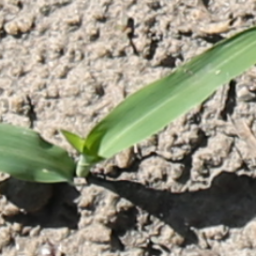

Supplement: S1 File — (ZIP) [file pone.0241528.s001.zip › S1-File/image/29.png]

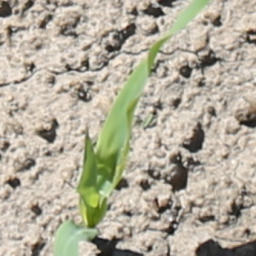

Supplement: S1 File — (ZIP) [file pone.0241528.s001.zip › S1-File/image/3.png]

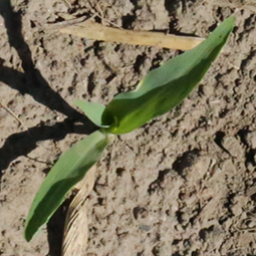

Supplement: S1 File — (ZIP) [file pone.0241528.s001.zip › S1-File/image/30.png]

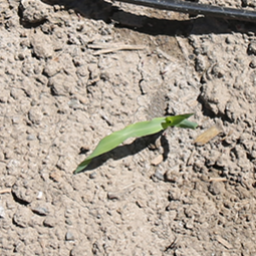

Supplement: S1 File — (ZIP) [file pone.0241528.s001.zip › S1-File/image/31.png]

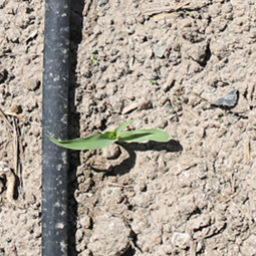

Supplement: S1 File — (ZIP) [file pone.0241528.s001.zip › S1-File/image/32.png]

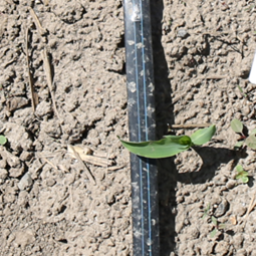

Supplement: S1 File — (ZIP) [file pone.0241528.s001.zip › S1-File/image/33.png]

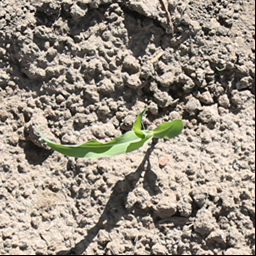

Supplement: S1 File — (ZIP) [file pone.0241528.s001.zip › S1-File/image/34.png]

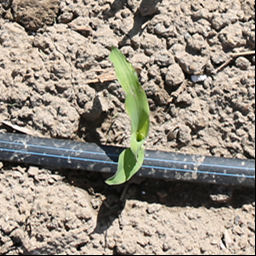

Supplement: S1 File — (ZIP) [file pone.0241528.s001.zip › S1-File/image/35.png]

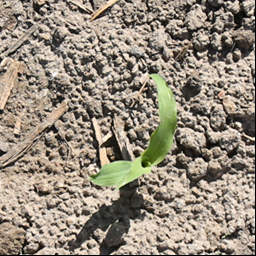

Supplement: S1 File — (ZIP) [file pone.0241528.s001.zip › S1-File/image/36.png]

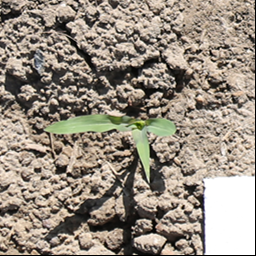

Supplement: S1 File — (ZIP) [file pone.0241528.s001.zip › S1-File/image/37.png]

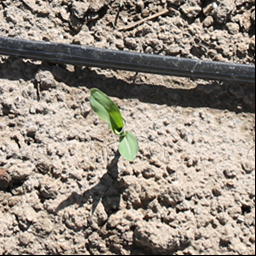

Supplement: S1 File — (ZIP) [file pone.0241528.s001.zip › S1-File/image/38.png]

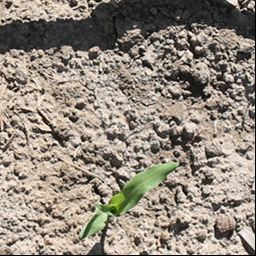

Supplement: S1 File — (ZIP) [file pone.0241528.s001.zip › S1-File/image/39.png]

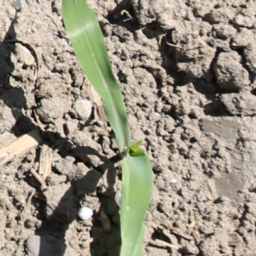

Supplement: S1 File — (ZIP) [file pone.0241528.s001.zip › S1-File/image/4.png]

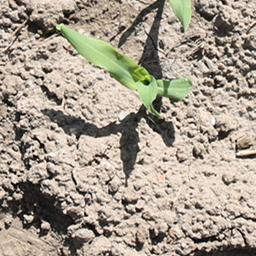

Supplement: S1 File — (ZIP) [file pone.0241528.s001.zip › S1-File/image/40.png]

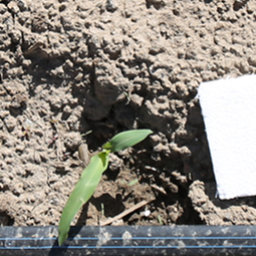

Supplement: S1 File — (ZIP) [file pone.0241528.s001.zip › S1-File/image/41.png]

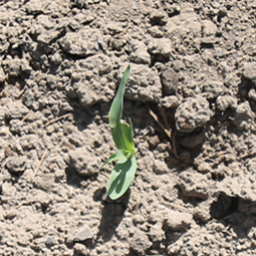

Supplement: S1 File — (ZIP) [file pone.0241528.s001.zip › S1-File/image/42.png]

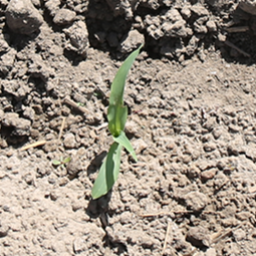

Supplement: S1 File — (ZIP) [file pone.0241528.s001.zip › S1-File/image/43.png]

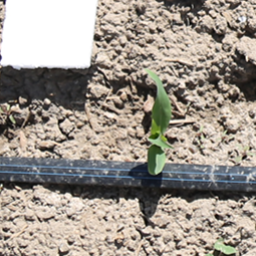

Supplement: S1 File — (ZIP) [file pone.0241528.s001.zip › S1-File/image/44.png]

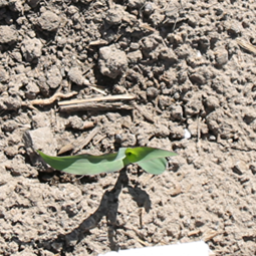

Supplement: S1 File — (ZIP) [file pone.0241528.s001.zip › S1-File/image/45.png]

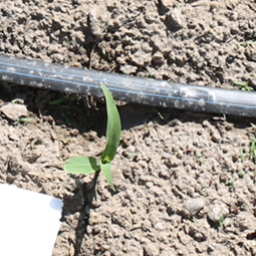

Supplement: S1 File — (ZIP) [file pone.0241528.s001.zip › S1-File/image/46.png]

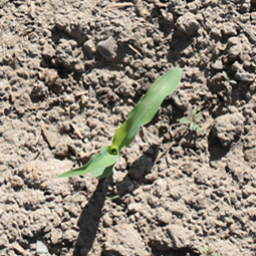

Supplement: S1 File — (ZIP) [file pone.0241528.s001.zip › S1-File/image/47.png]

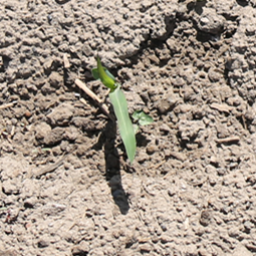

Supplement: S1 File — (ZIP) [file pone.0241528.s001.zip › S1-File/image/48.png]

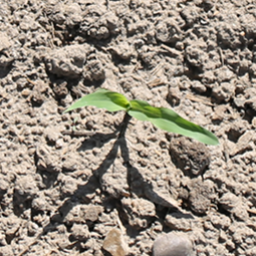

Supplement: S1 File — (ZIP) [file pone.0241528.s001.zip › S1-File/image/49.png]

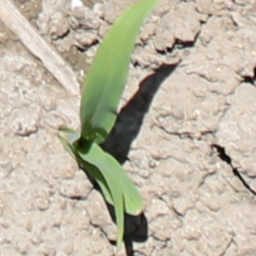

Supplement: S1 File — (ZIP) [file pone.0241528.s001.zip › S1-File/image/5.png]

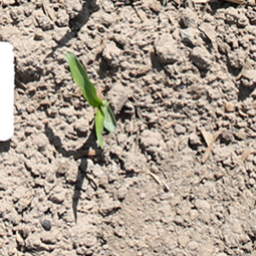

Supplement: S1 File — (ZIP) [file pone.0241528.s001.zip › S1-File/image/50.png]

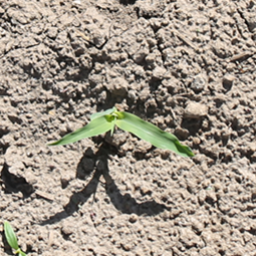

Supplement: S1 File — (ZIP) [file pone.0241528.s001.zip › S1-File/image/51.png]

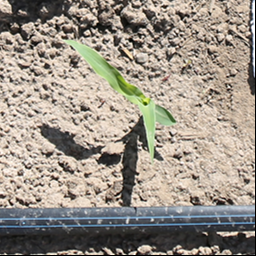

Supplement: S1 File — (ZIP) [file pone.0241528.s001.zip › S1-File/image/52.png]

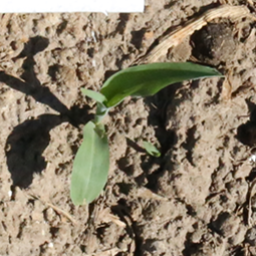

Supplement: S1 File — (ZIP) [file pone.0241528.s001.zip › S1-File/image/53.png]

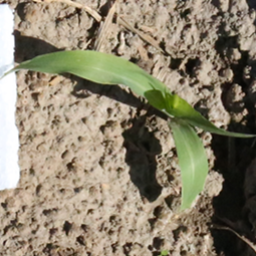

Supplement: S1 File — (ZIP) [file pone.0241528.s001.zip › S1-File/image/54.png]

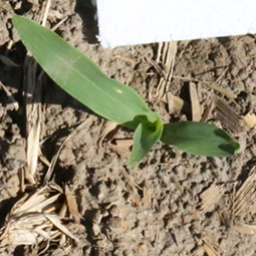

Supplement: S1 File — (ZIP) [file pone.0241528.s001.zip › S1-File/image/55.png]

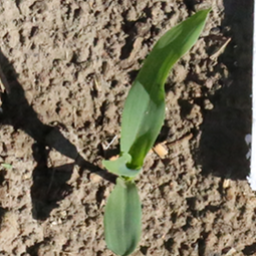

Supplement: S1 File — (ZIP) [file pone.0241528.s001.zip › S1-File/image/56.png]

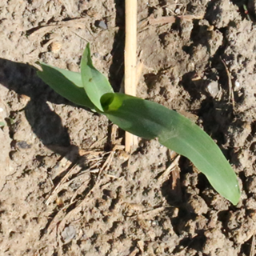

Supplement: S1 File — (ZIP) [file pone.0241528.s001.zip › S1-File/image/57.png]

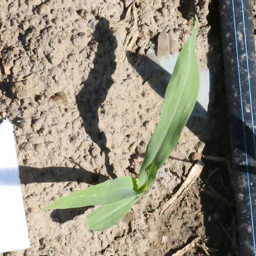

Supplement: S1 File — (ZIP) [file pone.0241528.s001.zip › S1-File/image/58.png]

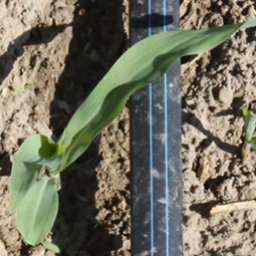

Supplement: S1 File — (ZIP) [file pone.0241528.s001.zip › S1-File/image/59.png]

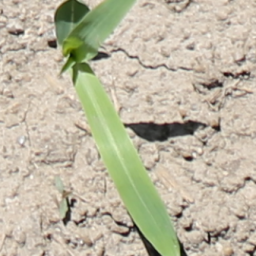

Supplement: S1 File — (ZIP) [file pone.0241528.s001.zip › S1-File/image/6.png]

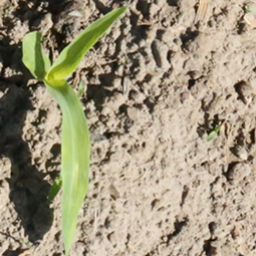

Supplement: S1 File — (ZIP) [file pone.0241528.s001.zip › S1-File/image/60.png]

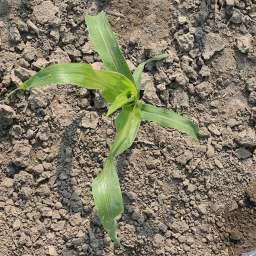

Supplement: S1 File — (ZIP) [file pone.0241528.s001.zip › S1-File/image/61.png]

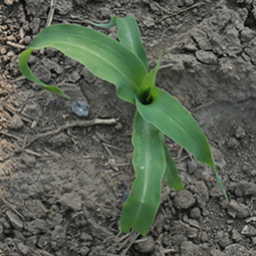

Supplement: S1 File — (ZIP) [file pone.0241528.s001.zip › S1-File/image/62.png]

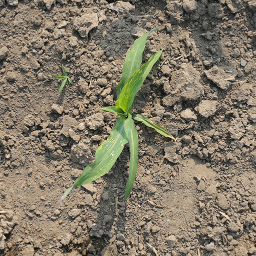

Supplement: S1 File — (ZIP) [file pone.0241528.s001.zip › S1-File/image/63.png]

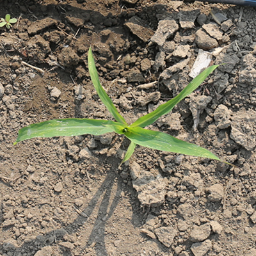

Supplement: S1 File — (ZIP) [file pone.0241528.s001.zip › S1-File/image/64.png]

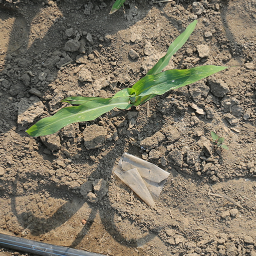

Supplement: S1 File — (ZIP) [file pone.0241528.s001.zip › S1-File/image/65.png]

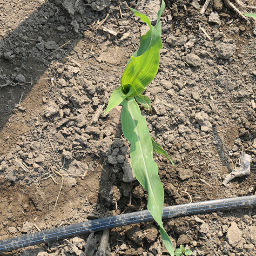

Supplement: S1 File — (ZIP) [file pone.0241528.s001.zip › S1-File/image/66.png]

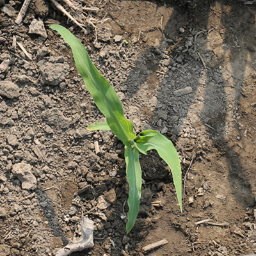

Supplement: S1 File — (ZIP) [file pone.0241528.s001.zip › S1-File/image/67.png]

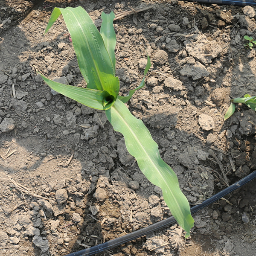

Supplement: S1 File — (ZIP) [file pone.0241528.s001.zip › S1-File/image/68.png]

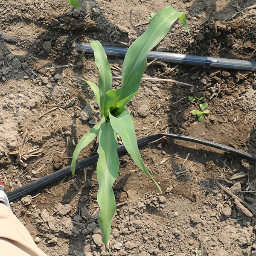

Supplement: S1 File — (ZIP) [file pone.0241528.s001.zip › S1-File/image/69.png]

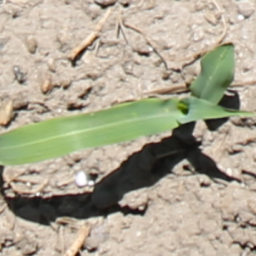

Supplement: S1 File — (ZIP) [file pone.0241528.s001.zip › S1-File/image/7.png]

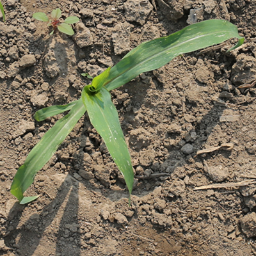

Supplement: S1 File — (ZIP) [file pone.0241528.s001.zip › S1-File/image/70.png]

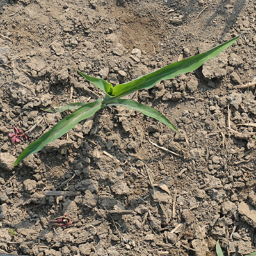

Supplement: S1 File — (ZIP) [file pone.0241528.s001.zip › S1-File/image/71.png]

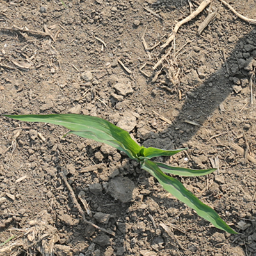

Supplement: S1 File — (ZIP) [file pone.0241528.s001.zip › S1-File/image/72.png]

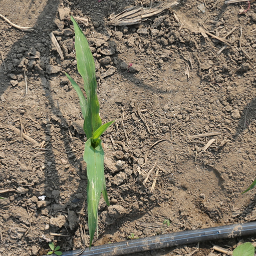

Supplement: S1 File — (ZIP) [file pone.0241528.s001.zip › S1-File/image/73.png]
