# Supplementary material for: High-throughput phenotyping analysis of maize at the seedling stage using end-to-end segmentation network
Source: PLoS One. 2021 Jan 12;16(1):e0241528. doi: 10.1371/journal.pone.0241528 (PMC7802938; doi:10.1371/journal.pone.0241528)
Supplement: Attachment — Submitted filename: note.pdf [file pone.0241528.s008.pdf]

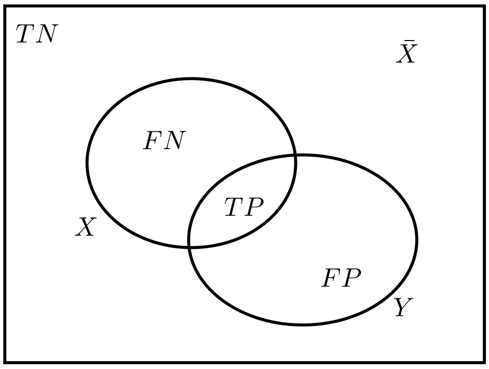

Figure 1: Key to symbols: X = positives ('ground truth'), $\bar{X}$ = negatives ('ground truth'), Y = predicted positives, TP = true positives, TN = true negatives, FP = false positives and FN = false negatives.

Theorem 1. *In a* one-class *segmentation problem the Sørensen-Dice coefficient (DSC) and the* F_1 *score are equivalent.*

Proof. By definition:

$$DSC = \frac{2\,|X \bigcap Y|}{|X| + |Y|} \tag{1}$$

where $|\cdot|$ indicates 'the cardinality of'. Now, with reference to Fig. 1, we have:

$$DSC = \frac{2TP}{2TP + FN + TP} \tag{2}$$

On the other hand, again by definition, we have:

$$F_1 = 2\frac{PR}{P + R} \tag{3}$$

where P and R respectively indicate *precision* and *recall*, i.e.:

$$P = \frac{TP}{TP + FP} \tag{4}$$

$$R = \frac{TP}{TP + FN} \tag{5}$$

From Eq. 4–5 we obtain:

$$P + R = \frac{TP\,(2TP + FP + FN)}{(TP + FN)\,(TP + FP)} \tag{6}$$

$$PR = \frac{TP^2}{(TP + FN)\,(TP + FP)} \tag{7}$$

Finally, substituting Eq. 6–7 into Eq. 3 we get:

$$F_1 = \frac{2TP}{2TP + FP + FN} \tag{8}$$

□
