# Supplementary material for: High-throughput phenotyping analysis of maize at the seedling stage using end-to-end segmentation network
Source: PLoS One. 2021 Jan 12;16(1):e0241528. doi: 10.1371/journal.pone.0241528 (PMC7802938; doi:10.1371/journal.pone.0241528)
Supplement: Attachment — Submitted filename: Response to Reviewers.docx [file pone.0241528.s006.docx]

Dear Editor,

We would like to submit our revised manuscript, titled “High-throughput phenotyping analysis of maize at the seedling stage using end-to-end segmentation network”, for publication in PLOS ONE. A 'Revised Manuscript with Track Changes' version of this revised manuscript is uploaded. Detailed corrections and responds to the reviewer’s comments are as follows:

**Reviewer #1s' comments:**

**1.  Page 26, Row 527: Most frequently in the article R2 was used; it is recommended to check, R or R2?**

Response: We checked the spelling in the article. R is used in reference [39], and is not a spelling error.

**2.  The entire References chapter needs to be revised and corrected according to the Submission Guidelines, PLoS ONE Journal, References section.**

Response: We carefully checked and revised the format of the references.

**Reviewer #2s' comments:**

**1.  It will be better to compare the proposed segmentation results with other recent segmentation methods and present it in the form of table.**

Response: Thanks for your comment. We added the comparison results of PlantU-net with other three recent segmentation methods according to the suggestions. For details, please see Table 3 and above contents in the revised version.

**2. It will be better if the recent papers in plant phenotyping are included as the references.**

Response: Thanks for your comment. We have cited these articles in the revised version.

Yinglun Li

College of Resources and Environment, Jilin Agricultural University, Changchun 130118, China; Tel: +86-10-5150-3362

Email: leeyinglun@163.com

13 Nov 2020
