# Supplementary material for: High-throughput phenotyping analysis of maize at the seedling stage using end-to-end segmentation network
Source: PLoS One. 2021 Jan 12;16(1):e0241528. doi: 10.1371/journal.pone.0241528 (PMC7802938; doi:10.1371/journal.pone.0241528)
Supplement: Attachment — Submitted filename: Response to Reviewers.docx [file pone.0241528.s007.docx]

Dear Editor：

Thank you for your valuable suggestions. Indeed, F1-Score and DICE (in Eq.7 and Eq.8) are two similar indicators. But in our opinion, they are different.

F1-score is an indicator to measure the accuracy of the two classification models. It takes account of the accuracy and recall of the classification model, which can be regarded as a weighted average of the accuracy and the recall. The Sørensen-Dice coefficient was usually used to estimate the similarity between the ground truth and the segmentation result.

We found that these two parameters were also used in similar articles to evaluate the accuracy of the research results [43].

We add a short explanation at the end of this section in the manuscript: “F1-Score and DICE are two similar but different indicators. F1-Score can be regarded as the weighted average of the accuracy and the recall of the model, while DICE is used to evaluate the similarity between the GT and the segmentation result [43].”

Looking forward to your reply！

[43] Praveen K, Domnic S. Rosette plant segmentation with leaf count using orthogonal transform and deep convolutional neural network. Machine Vision and Applications, 2020, 31:6. doi: 10.1007/s00138-019-01056-2.
