# Supplementary material for: High-throughput phenotyping analysis of maize at the seedling stage using end-to-end segmentation network
Source: PLoS One. 2021 Jan 12;16(1):e0241528. doi: 10.1371/journal.pone.0241528 (PMC7802938; doi:10.1371/journal.pone.0241528)
Supplement: Attachment — Submitted filename: Response to Reviewers.docx [file pone.0241528.s009.docx]

Dear Editor：

Thank you for your valuable suggestions.

After a detailed derivation, we found that the comment was indeed correct. The Sørensen-Dice Coefficient and the F1 score should give identical results. We have removed the duplicate evaluation metrics (The Sørensen-Dice Coefficient) in the revised version of this article.

At last, thanks again for your valuable comments and patient guidance.

Best regards！
